# VSV-G pseudotyping rescues HIV-1 CA mutations that impair core assembly or stability

**DOI:** 10.1186/1742-4690-5-57

**Published:** 2008-07-07

**Authors:** Sonia Brun, Maxime Solignat, Bernard Gay, Eric Bernard, Laurent Chaloin, David Fenard, Christian Devaux, Nathalie Chazal, Laurence Briant

**Affiliations:** 1 Université Montpellier 1, Centre d'études d'agents Pathogènes et Biotechnologies pour la Santé (CPBS), France; 2 CNRS, UMR 5236, CPBS, F-34965, Montpellier, France; 3 Université Montpellier 2, CPBS, F-34095, Montpellier, France; 4 GENETHON, 1bis rue de l'Internationale – BP60, 91002 EVRY cedex, France

## Abstract

**Background:**

The machinery of early HIV-1 replication still remains to be elucidated. Recently the viral core was reported to persist in the infected cell cytoplasm as an assembled particle, giving rise to the reverse transcription complex responsible for the synthesis of proviral DNA and its transport to the nucleus. Numerous studies have demonstrated that reverse transcription of the HIV-1 genome into proviral DNA is tightly dependent upon proper assembly of the capsid (CA) protein into mature cores that display appropriate stability. The functional impact of structural properties of the core in early replicative steps has yet to be determined.

**Results:**

Here, we show that infectivity of HIV-1 mutants bearing S_149_A and S_178_A mutations in CA can be efficiently restored when pseudotyped with vesicular stomatitis virus envelope glycoprotein, that addresses the mutant cores through the endocytic pathway rather than by fusion at the plasma membrane. The mechanisms by which these mutations disrupt virus infectivity were investigated. S_149_A and S_178_A mutants were unable to complete reverse transcription and/or produce 2-LTR DNA. Morphological analysis of viral particles and *in vitro *uncoating assays of isolated cores demonstrated that infectivity defects resulted from disruption of the viral core assembly and stability for S_149_A and S_178_A mutants, respectively. Consistent with these results, both mutants failed to saturate TRIM-antiviral restriction activity.

**Conclusion:**

Defects generated at the level of core assembly and stability by S_149_A and S_178_A mutations are sensitive to the way of delivery of viral nucleoprotein complexes into the target cell. Addressing CA mutants through the endocytic pathway may compensate for defects generated at the reverse transcription/nuclear import level subsequent to impairment of core assembly or stability.

## Introduction

The genome of the human immunodeficiency virus type 1 (HIV-1) is packaged within a conical shaped core formed by the viral capsid protein (CA) and delivered to the host cell cytoplasm upon fusion of the viral and cell membranes. Establishment of viral replication then requires the genomic RNA to be reverse transcribed into a double stranded proviral DNA. Upon completion of the reverse transcription (RT), full-length HIV-1 DNA associates into a functional pre-integration complex imported through the nuclear pore before integration into the host chromosome. Completion of HIV-1 RT appears to be a timely regulated process. Indeed, HIV-1 DNA synthesis is limited in intact viral core particles where late RT products are less efficiently synthesized than early DNA intermediates [1,2]. The synthesis of a complete viral DNA able to support efficient HIV-1 replication has formerly been assumed to depend on HIV-1 conical core disorganization and release of the reverse transcription complex (RTC) in the cell cytoplasm [3-5]. However, recent studies reported that CA may remain associated to the RTC in a ratio similar to that found in extracellular particles [6] and the presence of intact conical structures docked at the nuclear pore has been detected by electron microscopy imaging [7]. Accordingly, HIV-1 cores may not dissociate immediately after the viral fusion, but rather remain largely intact for at least a portion of the process from the initiation of RT to the synthesis of the central flap structure [7,8]. This model is further supported by the ability of RT to progress efficiently in intact virions, allowing the synthesis of full-length minus strand DNA in this core fraction, without requirement for an uncoating activity [2]. In this context, additional evidence for the persistence of assembled cores in the target cell has been provided through the ability of the tripartite motif (TRIM) family of antiviral factors to restrict HIV-1 replication in non-permissive cells through the recognition of a polymeric array of CA molecules present in intact cores [9-11]. The completion of viral DNA synthesis finally depends on the ability of the RTC to be addressed to an appropriate compartment of the infected cell. Indeed, drugs altering the integrity of the cytoskeleton [12] and RNA interference targeting the actin nucleator Arp2/3 complex [13] inhibit post-entry steps of the retroviral cycle. These data agree with imaging analysis in living cells showing that fluorescent HIV-1 complexes migrate as assembled cores along the actin cytoskeleton and microtubule network before being addressed to the microtubule-organizing center in the perinuclear region [6] or even to the nuclear pore itself [7] where uncoating may take place.

Mature HIV-1 cores are organized as a fullerene cone composed of a lattice of hexamerized CA protein [14,15]. According to structural studies, monomeric CA folds into two distinct globular domains: the N-terminal domain (NTD) (residues 1 to 145) and the C-terminal domain (CTD) (residues 151–231) are connected by a short flexible linker that folds in a 3_10 _helix upon oligomerization of CA [16-19]. Based on crystal structure data and cryoelectron microscopy reconstructions of soluble CA that spontaneously assembled into helical tubes and cones, models have been elaborated in which hexameric contacts at the NTD of adjacent CA drive the formation of the viral cores [15,20] while the CTD directs Gag-Gag precursor oligomerization between adjacent hexamers, linking and stabilizing hexameric rings into a continuous lattice [15,16,19,21]. Interactions were finally demonstrated between the NTD and CTD of adjacent hexamers that stabilize this network [20,21]. Mutational studies have widely demonstrated that synthesis of viral DNA and subsequent ability of HIV-1 to replicate into the host cells are tightly dependent upon the proper assembly and maturation of the viral core [22-25]. Moreover, the success of early post-entry events in the target cell requires an optimal stability of the incoming core [26]. This observation agrees with the existence of a fine regulation of the assembly/uncoating process. In this context, the possible contribution of post-translational modifications (i. e. phosphorylation) has been suggested as a candidate mechanism regulating the reversible nature of CA monomers interactions required for HIV-1 to assemble or disassemble core structures [27,28]. S_109_, S_149 _and S_178_, located in the NTD, the linker domain and the CTD of CA, respectively, have been identified as major phosphorylation sites in CA. Individual alanine substitutions at these positions were reported to abolish viral replication at early post entry steps [27]. However, the role of CA phosphorylation in virus replication is not clearly understood. In the present study, we took advantage of early post-entry defects reported for HIV-1 mutants bearing S_109_A, S_149_A and S_178_A substitutions in CA to investigate the functional role of the CA shell in early steps of replication. Based on saturation experiments performed in restrictive monkey cells, we found that all three mutants were unable to saturate TRIM-mediated restriction, indicating that they all display alterations in core structure. Elucidating the mechanisms by which these mutations disrupt virus infectivity, using biochemical and morphological analyses of viral particles and uncoating assays of envelope-stripped cores, demonstrated that alanine substitutions of S_149 _and S_178 _residues generated mild morphological defects or impaired stability of the core, respectively. S_109_A resulted in drastic alteration of core assembly and incomplete Gag precursor cleavage. Surprisingly, we found that when pseudotyped with the vesicular stomatitis virus glycoprotein (VSV-G), S_149_A and S_178_A, but not S_109_A, became competent for 2-LTR circle formation and established productive infection of the host cell. Altogether our data indicate that the appropriate shape and stability of the HIV-1 core are required for reverse transcription/nuclear import when delivered by fusion at the plasma membrane but dispensable when addressed through the endocytic pathway. In light of these results, we propose an additional function of the core in the HIV-1 life cycle, concerning replicative steps lying between the fusion event at the plasma membrane of the host cell and the integration of the viral genome.

## Results

### Infectivity of S_149_A and S_178_A mutants is restored when delivered to the host cell through the endocytic pathway

**Figure 1:**
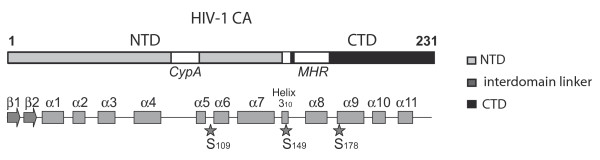
**Schematic representation of HIV-1 CA**. Location of α-helix and β-sheets identified in the N-terminal (NTD) and C-terminal (CTD) domain of CA is represented. The cyclophilin binding domain (CypA) and the Major Homology Region (MHR) are marked. Positions of S_109_, S_149 _and S_178 _residues are indicated.

**Figure 2:**
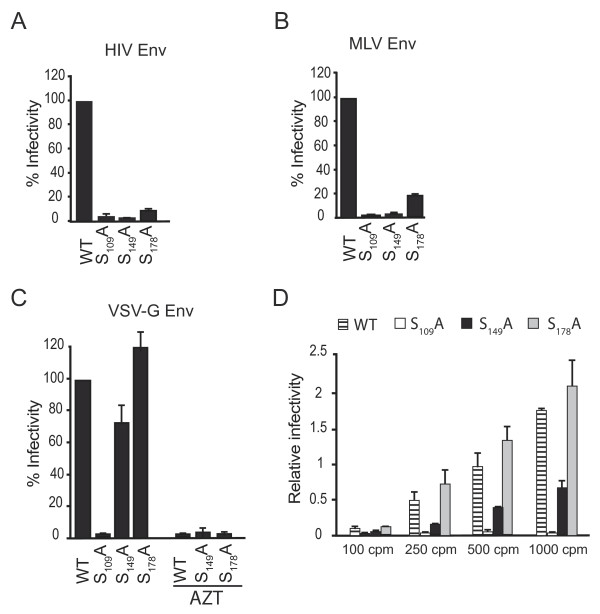
**Relative infection of pseudotyped CA mutant viruses**. Infectivity of normalized amounts (10,000 cpm RTase activity) of WT viruses and CA mutants expressing HIV-1 envelope (A) or having incorporated MLV (B) or VSV (C) glycoprotein was monitored using the MAGIC-5B indicator cell line. (C) Replication of VSV-G pseudotyped WT and CA mutants (10,000 cpm RTase activity) was inhibited by addition of 20 μM AZT to the culture medium. Values are expressed as a percentage of WT infectivity. (D) Rescue of CA mutant infectivity by VSV-G pseudotyping was investigated at low infectious doses (100 to 1,000 cpm RTase activity) by measuring o-nitrophenyl β-D-galactopyranoside hydrolysis in MAGIC-5B. Each value represents an average of three experiments performed in duplicate ± standard deviation.

The function of S_109_, S_149 _and S_178 _residues, positioned in the N-terminus, the interdomain linker and the C-terminus of CA respectively (see location in Figure 1), was analyzed using NL4.3 virions bearing an alanine substitution at each position. Viruses were produced by transfection experiments of 293T cells with pNL4.3 or S_109_A, S_149_A and S_178_A mutated molecular clones and used to infect the MAGIC-5B indicator cell line (Figure 2A). All three mutants were found to be poorly infectious compared to the NL4.3 wild-type (WT) viruses when viral input was normalized according to reverse transcriptase (RTase) activity. These data confirm replication defects previously reported for S_109_A, S_149_A and S_178_A mutants [27]. Infectivity of HIV-1 mutants characterized by post-entry blocks has been previously reported to be greatly influenced by the route of viral entry. Pseudotyping with envelope from other viruses enables some HIV-1 mutants to bypass early post entry blocks [29-31]. Incorporation of VSV-G, which allows viruses to enter the target cell using the endocytic low pH pathway, was found to restore infectivity of viruses bearing mutations/deletions in the Nef accessory gene [30] or of those lacking the ability to incorporate cyclophilin A (CypA) [31]. For testing similar effects, S_109_A, S_149_A and S_178_A mutants pseudotyped with VSV-G envelope were generated by co-transfection of proviral clones with a plasmid expressing VSV-G. VSV-G-pseudotyped S_109_A, S_149_A and S_178_A viruses (referred below as VSV-G-S_109_A, VSV-G-S_149_A and VSV-G-S_178_A respectively) were normalized for RTase content and used in single-round infectivity assays of the MAGIC-5B cell line (Figure 2C). As expected, when pseudotyped with VSV-G, a strong enhancement of infectivity was observed for WT virions as compared to non-pseudotyped viruses (data not shown). Interestingly, pseudotyping with VSV-G significantly restored infectious properties of S_149_A and S_178_A mutants to levels observed for VSV-G-WT viruses. In contrast, VSV-G-S_109_A viruses remained weakly infectious. Similar results were obtained using an extended range of viral input (Figure 2D and [Additional file 1]). At lower doses (100 to 1,000 cpm of RTase activity), VSV-G pseudotyped viruses did not saturated the cell culture, as less than 90% of the cells were found to be infected using a direct β-galactosidase staining assay (data not shown). Accordingly, the infectivity rescue observed for the VSV-G-S_149_A and VSV-G-S_178_A viruses cannot be ascribed to an overestimation due to the use of saturating doses of VSV-G-WT. MAGIC-5B cells were next infected with S_149_A or S_178_A viruses expressing either VSV-G or HIV Env using doses adjusted to generate comparable levels of strong-stop DNA copies in the infected cells as defined by qPCR experiments. In these cells, β-galactosidase activity was observed to be dramatically enhanced when S_149_A or S_178_A particles were delivered to the cell by the endocytic pathway [Additional file 2]. Finally, replication of VSV-G-S_149_A and VSV-G-S_178_A was abolished when MAGIC-5B cells were maintained in the presence of 20 μM AZT indicating that LTR-transactivation is not due to a pseudo-transduction artefact. Similar pseudotyping experiments were performed using the amphotropic murine leukaemia virus (MLV) glycoprotein, which allows infection of the target cell through a pH-independent fusion with the plasma membrane [32]. In these conditions, infectivity of MLV Env-S_109_A and MLV Env-S_149_A was comparable to that of the corresponding mutants expressing HIV Env. Indeed, β-galactosidase activity generated in MAGIC-5B cells was found to be 2% vs 4.3% for MLV Env-S_109_A and non pseudotyped S_109_A viruses respectively or 6% vs 4% for MLV Env-S_149_A and non-pseudotyped S_149_A viruses respectively when compared to WT virus expressing the corresponding envelope glycoproteins. (Figure 2A and 2B). Infectivity of S_178_A mutant was found slightly increased when pseudotyped with MLV Env (19% compared to 13% observed for MLV Env-S_178_A and non pseudotyped S_178_A viruses respectively). Infectivity defects associated with substitutions in CA can thus be rescued to variable extent by incorporation of different envelopes. Infectivity of S_149_A and S_178_A mutants was efficiently restored following VSV-G incorporation. Differences in infectivity observed between HIV Env expressing viruses and VSV-G pseudotypes suggest that shunting the viral core in the target cell through a pH-dependent endocytic pathway may bypass post-entry defects generated by mutation of S_149 _and S_178 _residues in CA.

### CA mutants display distinct behaviour during RT

**Figure 3:**
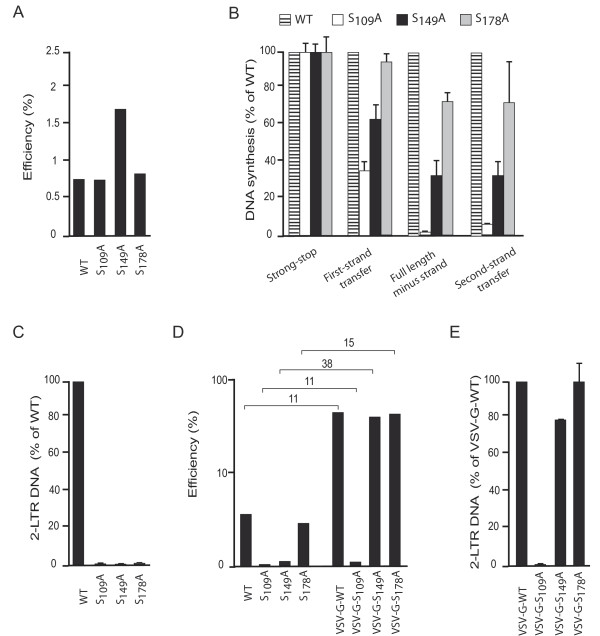
**Endogenous reverse transcription in cell free particles and proviral DNA synthesis in cells infected with WT or CA mutants expressing HIV Env or VSV-G**. (A) Competence for ERT of WT and mutant cell free particles after permeabilization and incubation with dNTPs. Efficiency of DNA synthesis is estimated as the ratio of second-strand transfer to strong-stop DNA detected by qPCR. Data presented are representative of three different experiments performed in duplicate. (B) MAGIC-5B cells were infected with normalized amounts of DNAse-treated WT, S_109_A, S_149_A or S_178_A viruses. Twenty-four hours post-infection, cells were lysed and RT intermediates were quantified by qPCR using primers specific for strong-stop or first-strand transfer or full-length minus strand or second-strand transfer DNAs. Results are the mean of three separate experiments performed in duplicate and are expressed as a percentage of values obtained with WT ± standard deviation. 2-LTR circle formation was detected by amplification of the LTR-LTR junctions in total DNA extracted from MAGIC-5B cells infected with WT or CA mutants expressing HIV Env (C) or pseudotyped with VSV-G (E). Results are the mean of three separate experiments performed in duplicate and are expressed as a percentage ± standard deviation of the values obtained with WT. (D) Efficiency of RT progression was evaluated in MAGIC-5B cells infected with WT or CA mutants expressing either HIV-Env or VSV-G. Values are expressed on a logarithmic scale and are representative of three separate experiments performed in duplicate. For each virus studied, RT efficiency in cells infected through VSV-G relative to that observed in cells infected with the corresponding virus expressing HIV-Env is indicated as a ratio.

Next, we examined the ability of CA mutants to accomplish early post entry events (i.e. RT). First of all, we tested whether mutations in CA impaired the RT machinery incorporated within viral particles using endogenous reverse transcription (ERT) experiments. This approach was successfully used to show alterations generated by CA mutations in Rous sarcoma virus [33]. Normalized amounts of cell free virions, determined by exogenous RTase activity, were permeabilized and incubated in the presence of dNTPs to promote DNA synthesis primed by the endogenous tRNA primer using the RNA genome as a template. Viral DNA synthesis was then monitored by qPCR detection of strong-stop and second-strand transfer DNAs. RT efficiency was established by calculating the fraction of second-strand transfer products generated relative to strong-stop DNA copies expressed as a percentage. As shown in Figure 3A, ERT activity occurred for all viruses tested indicating that early and late HIV-1 DNAs were produced at least as efficiently by CA mutants as WT viruses when viral cores were permeabilized through the use of detergent. Unexpectedly, ERT level was increased by 2.5 fold for the S_149_A mutant. Thus, alanine substitutions in CA did not markedly impair the formation of a functional nucleoprotein complex within the virions. We next analyzed RT capacities of CA mutants in the host cell. Total DNA was prepared from MAGIC-5B cells infected for 24 hours with normalized amounts of WT or CA mutant viruses and subjected to qPCR amplification using a specific set of primers allowing detection of RT intermediates. As shown in Figure 3B, strong-stop DNA was present at a similar ratio in cells infected with WT (2.9 × 10^6 ^copies/10^6 ^cells) or CA mutant viruses (from 2.7 to 3.9 × 10^6 ^copies/10^6 ^cells). All viruses tested were thus competent for fusion with the target cell membrane and inhibition of replication observed for S_109_A, S_149_A and S_178_A mutants occurred at a post-fusion step, as previously proposed [27]. Reverse transcription intermediates were efficiently detected in cells infected with WT virus (first-strand transfer, full-length minus strand and second-strand transfer DNAs were 8 × 10^5^; 2.5 × 10^5 ^and 2 × 10^5 ^copies/10^6 ^cells). In cells infected with the S_178_A mutant, first-strand transfer, full-length minus strand and second-strand transfer DNAs copies ranged from 70 to 95% levels quantified from cells infected by WT viruses. In contrast, RT was found most drastically altered in cells infected with S_149_A virus, as synthesis of full-length minus strand and second-strand transfer DNAs was reduced by nearly 70% compared to control conditions. Finally, level of first strand transfer DNA was significantly decreased and full-length minus strand DNA synthesis was almost abolished in cells infected with S_109_A viruses indicating that early reverse transcription was drastically reduced in these cells. We next tested the presence of 2-LTR circles in infected cells. 2-LTR circles are unproductive forms of viral DNA created by end-to-end ligation, that can be used as a reporter for nuclear import of the viral genome, since it localizes predominantly in the nucleus of infected cells [34]. Using primers that specifically amplify the LTR-LTR junction, we found that all CA mutants were impaired for 2-LTR circle formation (Figure 3C). Altogether, these data indicate that CA mutations impair early replication at different steps. S_109_A mutant was unable to accomplish RT. In contrast, S_149_A and S_178_A mutants produced different levels of second-strand transfer DNA and were impaired in their ability to produce 2-LTR circles.

### VSV-G pseudotyping of S_149_A and S_178_A mutants rescues 2-LTR circle formation

Having demonstrated that VSV-G incorporation rescues infectivity of S_149_A and S_178_A mutant particles, we investigated the ability of these pseudotyped mutants to synthesize proviral DNA. Strong-stop and second-strand transfer DNAs were quantified from infected cells by qPCR experiments and RT efficiency was calculated as described for ERT experiments (see above). Similar RT efficiency was observed in cells infected with VSV-G-S_149_A, VSV-G-S_178_A or VSV-G-WT viruses (Figure 3D). This indicates that RT progressed efficiently for these viruses. In contrast, RT progression remained dramatically impaired in cells infected with VSV-G-S_109_A viruses. These data were next compared to RT efficiency calculated from cells infected with viruses expressing HIV Env. Following VSV-G incorporation, RT efficiency was increased by 11 and 15-fold in cells infected with WT and S_178_A viruses respectively (Figure 3D). This stimulation was also observed for VSV-G-S_109_A, despite proviral DNA synthesis remaining dramatically inefficient. Interestingly, RT efficiency was enhanced by 38-fold when S_149_A viral particles contained VSV-G. Formation of 2-LTR DNA was next investigated in cells infected with VSV-G-S_149_A or VSV-G-S_178_A viruses (figure 3E). Consistent with infectivity assays, 2-LTR DNA was produced efficiently in cells infected with VSV-G-S_149_A and VSV-G-S_178_A. As expected, 2-LTR circles could not be amplified from cells infected with VSV-G-S_109_A. Altogether, our data indicate that infection through the endocytic pathway restored the ability of S_149_A and S_178_A mutants to produce 2-LTR DNA. Moreover, progression of RT from early to late steps was stimulated upon incorporation of VSV-G for all viruses studied, with a stronger effect observed for the S_149_A mutant. In conclusion, delivering the viral genome through a different route may enhance RT progression or possibly steps that allow nuclear import of the viral genome.

### Impact of S_109_A, S_149_A and S_178_A mutations on core recognition by TRIM family restriction factors

**Figure 4:**
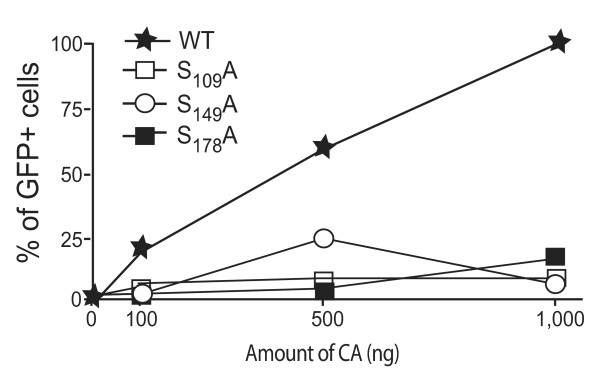
**TRIM5α saturation assay by WT or CA mutants**. Cos7 cells incubated with increasing amounts of VSV-G pseudotyped WT or S_109_A, S_149_A or S_178_A-mutated virions were challenged with fixed infectious doses of HIV-1_R7_-GFP indicator virus. The percentage of replication (GFP positive cells) was quantified by flow cytometry at day 2 post challenge. Values are plotted as the percentage of GFP positive cells as a function of the amount of VSV-G pseudotyped virus used for saturation of TRIM5α proteins. Data presented are representative of two different experiments.

We next examined the capacity of S_109_A, S_149_A and S_178_A mutants to saturate the TRIM family proteins. TRIM proteins expressed in some Old World monkey cells efficiently restrict post-entry steps of HIV-1 replication [9,10]. While the precise mechanisms of this restriction remain unclear, the restrictive properties of TRIM factors are correlated with their capacity to interact with the incoming viral cores [35,36]. TRIM recognition is sensitive to the maturation of the CA-p2-NC junctions, to the stability of the capsid shell and finally to the organization and folding of exposed surfaces of the assembled core [11,36,37]. We thus took advantage of these characteristics to evaluate the impact of S_109_A, S_149_A and S_178_A substitutions on structural properties of the corresponding cores. HIV-1 infection in Cos7 cells is restricted by TRIM5α. This restriction can be saturated in the presence of virus-like particles or elevated amounts of saturating virus that displays properly folded cores with an appropriate stability [38]. To evaluate the impact of S_109_A, S_149_A and S_178_A mutations on core integrity, Cos7 cells were infected with increasing amounts of VSV-G pseudotyped WT or S_109_A or S_149_A or S_178_A virions and challenged with a fixed amount (200 ng CA protein defined by anti-CA ELISA assay) of an HIV-1 reporter virus bearing a GFP sequence in place of the *nef *gene (HIV-1_R7_-GFP) [39]. This dose was previously ascertained to allow expression of GFP at less than 1% in OMK cells (data not shown). When cells were co-infected with increasing amounts of pseudotyped WT virus, an increasing percentage of GFP-positive cells could be detected by FACS analysis indicating a dose-dependent enhancement of HIV-1_R7_-GFP reporter virus replication (Figure 4). When similar experiments were performed saturating Cos7 cells with increasing amounts of S_109_A, S_149_A, S_178_A viruses, GFP expression levels remained extremely reduced, indicating that TRIM5α-mediated restriction of HIV-1_R7_-GFP replication could not be overcome by the presence in the infected cells, of cores bearing S_109_A, S_149_A or S_178_A mutations. Similar results were obtained using the OMK cell line that expresses the TRIMCyp restriction factor, which results from a retrotransposition event of the cyclophilin A pseudogene into the TRIM5 locus [10,40] (data not shown). In conclusion, CA mutants were thus poorly recognized by TRIM5α in Cos7 cells or TRIMCyp in OMK cells. As TRIM recognition of the incoming viral cores requires the proper intermolecular packing of adjacent CA molecules, our results indicate that substitution of serine residues in CA generates modifications in the capsid shell that render viral cores resistant to the cellular restriction machinery.

### In vivo assembly and maturation of S_109_A, S_149_A and S_178_A mutant viruses

**Figure 5:**
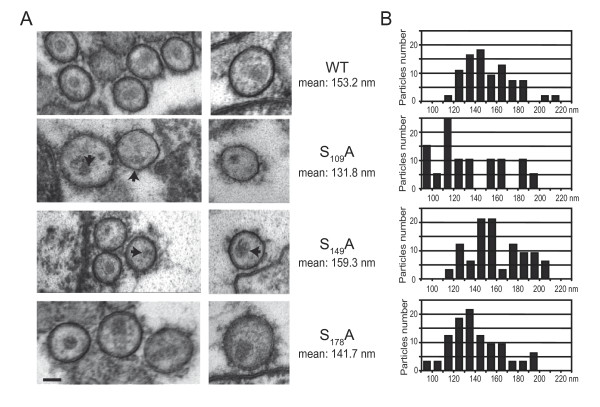
**Analysis of WT and mutants virion morphology**. (A) Virus particles produced from 293T cells transfected with WT, S_109_A, S_149_A or S_178_A molecular clones were processed as described in "Materials and Methods" and imaged in thin-layer electron microscopy. Aberrant cores are indicated by an arrow. Identical scale is used for all images (scale bar = 60 nm). (B) Diameter distribution observed for WT and mutant viruses. Mean diameters are indicated.

The effect of CA mutations on Gag assembly was investigated by analyzing cell free viral particles morphology in electron microscopy experiments. As shown in Figure 5A and 5B, the presence of normal-sized mature virions was observed in preparations of WT, S_149_A and S_178_A particles. In contrast, S_109_A particles displayed a significant decrease in size. No modification of the sphericity index was observed for any mutant tested (data not shown). The presence of unambiguous cone-shaped nucleoids was observed in WT and S_178_A viruses. In contrast, S_149_A particles displayed mild morphological defects characterized by irregularly shaped cores. When S_109_A viruses were examined, no core was observed and viruses contained aggregates that were acentric and presented a lobular aspect.

**Figure 6:**
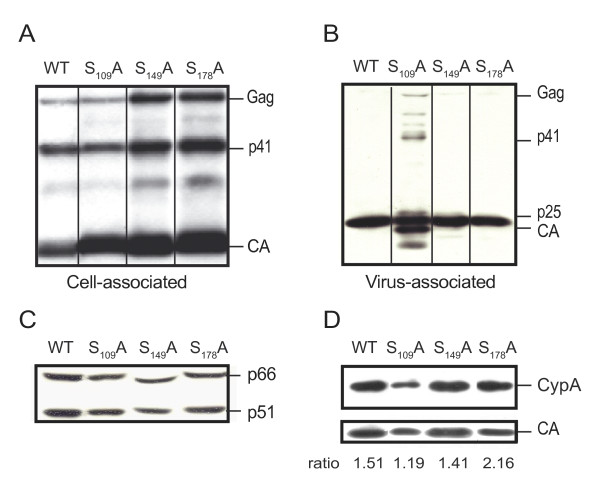
**Western-blot analysis of cell free virions and Gag expression in cell lysates**. 293T cells were transfected with WT or derived CA mutant HIV-1 molecular clones. Cell-associated proteins (A) or normalized amounts of viral particles present in culture supernatant (B) were processed for Western blotting experiments as described under "Materials and Methods" and revealed with anti-CA mAbs. Position of unprocessed Gag, p41 and p25 processing intermediates and mature CA is indicated. (C) Expression and maturation of the RTase subunits were determined from pelleted cell-free virions using anti-RTase mAbs. (D) Incorporation levels of CypA into WT and mutant virions were determined using anti-CypA. The mean CypA to CA ratio determined by densitometry scanning from three different experiments is indicated for each lane.

The Gag expression and processing pattern was further characterized at the level of cell-associated proteins and cell free particles in immunoblotting experiments. When viral lysates of 293T transfected cells were reacted with anti-CA mAbs, expression and processing of the Gag structural precursor were found to be similar for cells expressing WT and mutants viruses (Figure 6A). Analyzing cell free virions normalized according to RTase activity (Figure 6B), the expected ratio of Gag precursor, p41 intermediate and mature CA were observed for S_149_A and S_178_A virions as compared to WT. In contrast, S_109_A virions presented an accumulation of unprocessed Gag and processing intermediates, including p41 and p25. Hence, Gag maturation is compromised by S_109_A mutation in CA. All mutant viruses were found to package processed Pol (p51 and p66) proteins at a normal level relative to WT virions (Figure 6C). Finally, no significant difference in the levels of CypA incorporation was detected from WT and CA mutants (Figure 6D). Altogether these data indicate that the S_178_A mutation does not compromise the ability of CA to assemble *in vivo*. The S_149_A mutation generates subtle core morphological defects despite efficient maturation of CA. Finally, production of viral particles with normally assembled and mature cores strictly depends upon S_109 _integrity.

### Contribution of S_109_, S_149 _and S_178 _residues to HIV-1 core stability

**Figure 7:**
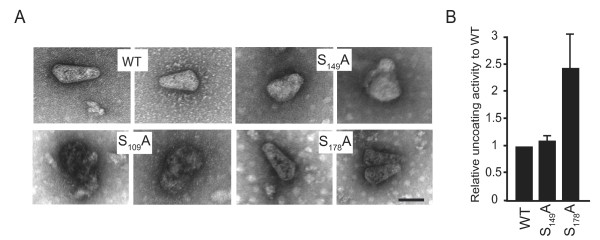
**Analysis of WT and mutants core morphology and stability**. (A) Envelope of WT and CA mutants was removed by detergent treatment and isolated cores were analysed by negative-stain electron microscopy. Electron microscopy pictures shown are representative of observations performed on 20 to 60 cores. The scale is indicated (90 nm) and is identical for all images shown. (B) Uncoating activity of WT and mutant cores. Results are normalized according to uncoating activity of WT cores. The values represent an average of at least two experiments ± the standard deviation.

Stability of WT and mutant cores was addressed using an *in vitro *dissociation assay. The corresponding cell-free particles were envelope-stripped by ultracentrifugation through a sucrose cushion overlaid with detergent as described in Materials and Methods. Preparations of WT cores processed for negative staining and electron microscopy revealed recognizable intact, cone-shaped particles with a small fraction of unstructured aggregates and spherical structures, typical of CA monomers (Figure 7A). Cone shaped structures were also predominantly observed from preparations of S_178_A cores and more rarely from S_149_A samples. On the contrary, aberrant aggregates, but no core, were isolated from S_109_A mutants. These results are consistent with data obtained from the biochemical and morphological analyses of entire S_109_A particles. Due to the aberrant morphology of the structures purified, the S_109_A mutant was excluded from subsequent experiments. Preparations of WT, S_149_A and S_178_A cores were then incubated for 2 h at 37°C in conditions that were previously reported to allow core destabilisation [26]. Uncoating efficiency was determined after centrifugation of the reaction mixture and a measure of CA concentration in the soluble (monomeric CA) and pellet fractions (assembled cores) was made using an anti-CA ELISA assay. In these experimental conditions, incubation at 37°C efficiently promoted dissociation compared to incubation at 4°C (data not shown). When mutant cores were assayed, no significant variation in uncoating efficiency was observed for S_149_A cores compared to WT (Figure 7B). Similar incubation of S_178_A core particles revealed an increased dissociation rate. Thus alanine substitution of the S_178 _residue in CA decreases the stability of HIV-1 cores.

## Discussion

Earlier observations have identified S_109_, S_149 _and S_178 _residues as major phosphoacceptor sites in CA [27]. While their precise contribution to HIV-1 replicative capacity remains to be defined, each residue was previously reported to be required for viral replication at the level of post entry steps [27]. Here we took advantage of this phenotype to study the relationships that exist between structural properties of the core and early post-entry events of HIV-1 infection. Analyzing key steps of the viral life cycle, we demonstrate that all three residues are crucial for the formation of properly assembled HIV-1 cores and deal with distinct functions, including Gag precursor maturation, mature CA assembly, or stabilizing the assembled core structure, for S_109_, S_149 _and S_178 _residues respectively. We confirm that alanine substitution at each site impairs HIV-1 replication during early post-entry steps, and we further demonstrate that replication blocks occur at different steps of the RT/nuclear import process. Unexpectedly, replication defects could be efficiently overcome by pseudotyping S_149_A and S_178_A viral particles with VSV-G, which was found to enhance late DNA synthesis and/or 2-LTR circle production. These results indicate that the impact of structural properties of HIV-1 cores on post-fusion events is sensitive to the way of delivery of the HIV-1 core to the target cell.

Electron microscopy analyses and biochemical experiments did not reveal any morphological or maturation defect in the S_178_A mutant. This mutant was, however, unable to saturate the TRIM5α/TRIMCyp restriction factors. Moreover, the corresponding envelope stripped cores dissociated *in vitro *more rapidly than WT cores, indicating that the loss of infectivity and post-entry blocks observed for the S_178_A mutant correlate to modifications in *in vitro *core stability. The S_178 _residue lies at the beginning of α-helix 9 involved both in CTD dimerization and N- to C-domain intersubunit interactions that are crucial for the formation of the CA lattice [16,19,20]. According to structural models, the presence of a neutral side chain at position S_178 _was proposed to participate in an electrostatic intersubunit repulsion of the CTD domains maintaining an appropriate stability of the CA lattice by increasing the inter-hexamer distance [41]. Our data, confirming these models, agree that the integrity of the S_178 _residue provides a stabilizing effect on the structure of the assembled core. Proviral DNA analysis revealed that the S_178_A mutant was only slightly affected in synthesis of the different DNA intermediates checked. However, 2-LTR production was completely impaired. This phenotype is very reminiscent of that reported by the Aiken laboratory for the Q_63_A/Q_67_A double mutant that displayed an unstable core structure and which was competent for RT [26,42]. Considering the S_178_A mutant, stability impairment may be deleterious for steps lying between the final RT and integration (i.e. formation of LTR ends or viral DNA nuclear import). It must be considered that a nuclear import defect may account for the lack of infectivity observed. Relationships that exist between core organization/stability and early replicative steps still remain unclear. However, it is conceivable that structural properties of the core may modulate RT and/or subsequent steps. Indeed, modifications in core morphology are observed during the RT process [3]. In contrast, the efficiency of RT is enhanced in reorganizing the core through the use of detergent [2]. Such modifications may however be subtle since the presence of intact cores has recently been detected in the cytoplasm of infected cells [6,43,44]. Altogether, these observations support that the persistence of the CA shell in an appropriate state, while part or all of the RT step, is required for early post-entry steps.

Mutation of the S_149 _residue also resulted in a profound defect in virus infection of MAGIC-5B cells. Maturation and stability of the assembled mutant cores were unaffected *in vitro *and no defect could be identified using biochemical approaches. Only slight morphological changes were revealed from electron microscopy imaging of cell free S_149 _viruses and isolated cores. The lack of infectivity observed for this mutant may thus result from subtle defects in CA assembly. This hypothesis was further confirmed using saturation assays of TRIM restriction factors performed in Cos7 monkey cells, as S_149_A viruses failed to release TRIM-mediated restriction observed in these cells. This phenotype is consistent with the location of S_149 _within the flexible linker connecting the N- and C-terminal domain of CA which contributes to retroviral core assembly, probably through the stabilization of dimeric CA contacts [16]. Analyzing proviral DNA synthesis, S_149_A was found altered at the level of first-strand transfer DNA and subsequent steps that resulted in abolition of 2-LTR formation. Such defects may result from the inappropriate assembly of the core but are not related to instability of the CA shell.

It is evident from our study that replication defects generated by alanine substitutions of S_149 _and S_178 _residues can be overcome when viral particles are pseudotyped with VSV-G. Indeed, VSV-G-S_149_A and VSV-G-S_178_A efficiently supported RT and 2-LTR circle formation. Furthermore, VSV-G pseudotyping was found to enhance efficiency of late RT in cells infected with the S_149_A mutant. Interestingly, S_149_A and S_178_A mutants were not or poorly rescued by incorporation of MLV Env that triggers the viral core into the host cell cytoplasm by fusion at the plasma membrane. Distinct defects in proviral DNA production, associated with mutations that modify core assembly or stability, are thus circumvented by targeting virus entry to a different compartment of the cell. Pathways that may route the nucleoprotein viral complexes after fusion and delivery to the host cell are not clearly defined. An increasing amount of evidence suggests that the cytoskeleton of the target cell facilitates early steps of HIV-1 infection. Tracking fluorescent viral complexes in living cells infected with VSV-G pseudotyped virus demonstrated that short distance rapid movements of HIV-1 cores are characteristic for an actin-polymerization-dependent transport [6,44]. The same studies reported that the microtubule network supports long distance movement of HIV-1 core complexes. In cells infected with non pseudotyped HIV-1, RT is impaired by the use of an actin-depolymerization agent [12]. However, actin-cable-dependent trafficking systems recruited by HIV-1 complexes delivered through HIV Env and VSV-G may differ. Indeed, HIV-1 infection is blocked at the RT level in cells expressing siRNAs to the Arp2/3 actin nucleator complex, which inhibits polymerization of actin. This block is no longer observed using VSV-G pseudotyped HIV-1 [13]. Here, we found that VSV-G incorporation restores infectivity of S_149_A and S_178_A mutants. Accordingly, the relationships between the different phenotypes observed upon incorporation of HIV-Env and VSV-G and the recruitment of different trafficking pathways in the host cell must be considered. In this context, core defects reported herein for S_149_A and S_178_A mutants may affect the capacity of the viral reverse transcription complex to relocalize to an appropriate site in the host cell cytoplasm. The ability of S_178_A and S_149_A cores to traffic into the target cell after viral fusion, and the characterization of reverse transcription/preintegration complexes formed in the infected cells, would provide further understanding in the contribution of the CA shell during HIV-1 post-entry steps.

In contrast, the restoration of infectious properties was not observed for S_109_A virions pseudotyped with VSV-G. This phenotype correlates with an early and total block in first-strand transfer DNA synthesis observed in cells infected with the S_109_A mutant. Furthermore, S_109_A substitution was found to dramatically impair Gag precursor processing and *in vivo *core assembly. Thus, this study does not confirm a previous report by Wacharapornin et al. [28] stating that assembled cores may be purified from S_109_A mutated viruses. Impairment of core assembly and maturation defects observed from this mutant agree with the position of the S_109 _residue at a proximity of α-helix 6, an assembly sensitive surface required for core formation [23] that undergoes spatial rearrangements during capsid assembly [45]. Interestingly, a conserved serine residue is present at the corresponding position in the SIV capsid protein. Substitution of this amino-acid with alanine resulted in mild processing defects in cell free virions [46], a phenotype reminiscent of that reported for the HIV-1 S_109_A mutant in the present study.

A question that remains to be addressed is the potential contribution of post-translational modifications in regulating CA assembly, maturation and stability. S_109_, S_149 _and S_178 _residues have previously been identified as major phosphorylation sites in CA [27]. Recent reports showed that incoming HIV-1 cores, accumulated as stable complexes in the cytoplasm of quiescent T lymphocytes, support viral gene expression upon serum stimulation of the host cell [43]. Furthermore, *in vitro *dissociation of HIV-1 cores is stimulated by addition of cell extract prepared from activated T lymphocytes [47]. Very recently, mutations in CA were found to inhibit HIV-1 replication in non-dividing cells in a cell-type dependent fashion, suggesting that CA may be the target of cellular factors regulating the role of incoming viral cores in target cells [48]. According to these data, the regulation of the shape/stability of HIV-1 CA by cellular factors may determine the trafficking ability of incoming viral cores. The contribution of cellular kinases, the presence of which has been detected in purified HIV-1 particles, [49-52] has to be considered in this context. Analyzing the dynamics of CA phosphorylation during Gag processing, assembly of mature CA and early post-fusion steps may provide a crucial insight into early replicative steps.

## Materials and methods

### Plasmids

pNL4.3 and pR7-GFP HIV-1 molecular clones [39], pHEF-VSV-G [53] and SV-A-MLV-env [54] vectors encoding envelope glycoproteins of VSV and MLV, respectively, were obtained through the AIDS Research and Reference Reagent Program, NIAID, NIH. pNL4.3_S109A_, pNL4.3_S149A_, and pNL4.3_S178A _molecular clones have been described elsewhere [27].

### Viral stock production

Viruses were produced by transfection of 293T cells with HIV-1 molecular clones using the JetPei transfection reagent (QBiogen). Pseudotyping of HIV-1 viruses was achieved by co-transfection with the pHEF-VSV-G or the SV-A-MLV-env plasmid. Two days after transfection, virus-containing supernatants were collected, filtered onto 0.22 μM membranes, aliquoted and stored at -80°C.

### Infectivity assays

MAGIC-5B indicator cells, which stably express the β-galactosidase reporter gene cloned downstream of the HIV-1 LTR promoter, were plated at 8 × 10^4 ^cells per well, in 24-well plates and exposed to HIV-1 stock solutions normalized according to RTase activity determined as previously reported [55]. Forty-eight hours post-infection, viral infectivity was monitored by quantification of o-nitrophenyl β-D-galactopyranoside hydrolysis from cell lysates as previously described [55]. β-galactosidase activity was evaluated by measuring absorbance at 405 nm and was normalized according to total protein content in the cell lysate.

### PCR analysis of viral DNA in infected cells

Total DNA was extracted from MAGIC-5B cells (8 × 10^4^) infected for 24 h with normalized amounts (4.5 × 10^4 ^cpm RTase activity) of DNAseI-treated virus. The presence of contaminating pNL4.3 plasmid DNA was checked as previously described [27]. HIV-1 DNA synthesis was then monitored by qPCR as follows: 100 ng total DNA sample were added to the reaction mix containing 0.4 μM of each primer, and 2 μl SYBR Green master amplification mix (Fast start DNA Master plus SYBR Green I amplification kit, Roche). For each amplification, a control reaction was performed in which DNA sample was replaced by water. Reactions were subjected to a first cycle of 10 min at 95°C followed by 40 amplification cycles of 15 s at 95°C; 15 s at 65°C and 20 s at 72°C on the RotorGene system (Labgene). Fluorescence signal was recorded at the end of each cycle. A standard curve was generated from 10 to 100,000 copies of pNL4.3 plasmid. The copy numbers of HIV-1 DNA were normalized to that of the GAPDH DNA quantified in parallel as an endogenous control. Primers used for amplification were: strong-stop DNA: 5'-AAGCAGTGGGTTCCCTAGTTAG-3' and 5'-GGTCTCTCTGGTTAGACCA-3'; first-strand transfer: 5'-AGCAGCTGCTTTTTGCCTGTACT-3' and 5'-ACACAACAGACGGGCACACAC-3'; full-length minus strand, 5'-CAAGTAGTGTGTGCCCGTCTGTT-3' and 5'-CCTGCGTCGAGAGAGCTCCTCTGG-3' and second-strand transfer 5'-AGCAGCTGCTTTTTGCCTGTACT-3' and 5'-CCTGCGTCGAGAGAGCTCCTCTGG-3'. Amplification of 2-LTR circles was performed as previously reported [56].

### Endogenous reverse transcription assay

Sucrose cushion-purified virus particles (normalized according to exogenous RTase activity to 4.5 × 10^4 ^cpm) were incubated for 18 h at 37°C in the presence of 0.2 mM Triton X-100, 10 mM Tris, pH 7.4, 10 mM MgCl_2_, and 200 μM of each dNTP in a final volume of 50 μl. A no-nucleotide control reaction mixture was always included. Nucleic acids were then extracted once with phenol:chloroform:isoamyl alcohol (25:24:1) and once with chloroform, and precipitated with ethanol. Purified products were resuspended in 50 μl of water and used (2.5 μl) in qPCR experiment as described above.

### TRIM saturation assay

5 × 10^4 ^OMK or Cos7 cells were plated in 24-well plates the day before infection. The cells were co-infected with VSV-pseudotyped HIV-1 or CA mutant particles and superinfected 3 hours later with an appropriate dose (200 ng CA) of VSV-pseudotyped HIV_R7_-GFP reporter virus. Two days after inoculation, cells were detached using trypsin and analyzed for GFP expression using an EPICS PROFILE XL4C cytometer (Beckman Coulter). Values were reported as the percentage of GFP+ cells in the culture. VSV-G-pseudotyped HIV-GFP reporter particles were titrated onto each cell line to determine the appropriate dose for use in the restriction assay.

### Electron Microscopy Analysis

Virus-producing cells were processed for thin-layer electron microscopy as described elsewhere [55]. For negative staining, 20 μl sample core suspensions were applied to Formvar-coated grids (mesh size, 200) and stained with 2% uranyl acetate for 1 min. Preparations were examined with a Hitachi H.7100 transmission electron microscope.

### Immunoblotting analysis

Cell or cell-free virions pelleted by ultracentrifugation (350,000 g, 5 min at 4°C) were solubilized in SDS-PAGE sample buffer and separated on a 12.5% SDS-PAGE. Proteins transferred to PVDF membrane (Millipore) were revealed using a rabbit polyclonal serum directed to RT (kindly provided by J. L. Darlix, ENS, Lyon, France) or CypA (Biomol Research Laboratories Inc.), and mAbs raised against CA (IOT34A clone; Abcam) or actin (C4 clone; MP Biomedicals). Secondary antibodies conjugated to horseradish peroxidase were revealed by enhanced chemiluminescent detection (Pierce Biotechnology, Inc.).

### Core isolation and *in vitro *uncoating assay

Viral cores were purified by a spin-through technique. Briefly, cell free virions were loaded onto the top of a discontinuous sucrose density gradient composed of 1 ml 50% sucrose at the bottom covered by 1 ml Triton 0.1% in 10% sucrose and centrifuged at 100,000 g in a SW50.1 rotor (Beckman) for 2 h at 4°C. Cores were then resuspended in PBS and stored at -80°C. *In vitro *uncoating was assayed by incubating purified cores diluted in 10 mM Tris HCl pH 7.4, 100 mM NaCl, 1 mM EDTA for 2 h at 4°C or at 37°C. After ultracentrifugation for 20 min at 13,800 g, 4°C, the extent of core dissociation was calculated by the ratio of CA detected in the supernatant (oligomeric CA) to that present in pellets (assembled cores) by anti-CA ELISA assay.

## Abbreviations

AZT: Azidothymidine; CA: Capsid protein; CTD: C-terminal domain; CypA: Cyclophilin A; ERT: Endogenous reverse transcription; HIV-1: Human immunodeficiency virus type 1; MLV: Murine leukemia virus; NTD: N-terminal domain; qPCR: Quantitative PCR; RT: Reverse transcription; RTase: Reverse transcriptase; RTC: Reverse transcription complex; TRIM: Tripartite motif; VSV-G: Vesicular stomatitis virus glycoprotein; WT: Wild-type.

## Competing interests

The authors declare that they have no competing interests.

## Authors' contributions

SB, MS, and EB performed the experiments, BG performed the electron microscopy studies, NC, CD and LC contributed to experiments, analyzed the data and participated in manuscript redaction, LB conceived and designed the experiments as well as wrote the paper. All authors read and approved the final manuscript.

## Supplementary Material

Additional file 1 **Infectivity of increasing amounts of WT viruses and CA mutants expressing HIV Env or VSV-G**. Viral inputs were normalized according to RTase activity (ranging from 100 to 10,000 cpm) and used to infect MAGIC-5B cells. 48 hours post-infection, relative infectivity was measured by quantification of o-nitrophenyl β-D-galactopyranoside hydrolysis. Time of incubation used for revelation of β-galactosidase assay was adapted to produce non saturating OD at 405 nm (1h45 in panel A and 25 min in panel B). For each dose tested, infectivity of S_149_A and S_178_A mutants is indicated as a percentage of β-galactosidase activity generated by an identical amount of WT viruses with the corresponding envelope. Click here for file

Additional file 2 **Quantification of β-galactosidase activities generated in MAGIC-5B cells infected with amounts of CA mutants expressing HIV Env or VSV-G that generated comparable strong-stop DNA copy numbers**. MAGIC-5B cells were infected with S_149_A or S_178_A mutants expressing HIV Env or VSV-G, using infectious doses that generated similar strong-stop DNA copy numbers as measured by qPCR 24 h post-infection. Relative infectivity is expressed as o-nitrophenyl β-D-galactopyranoside hydrolysis measured from total cell extracts by absorbance at 405 nm. Each value represents an average of two experiments performed in duplicate ± standard deviation. Click here for file
