# Supplementary figures and images for: VSV-G pseudotyping rescues HIV-1 CA mutations that impair core assembly or stability
Source: Retrovirology. 2008 Jul 7;5:57. doi: 10.1186/1742-4690-5-57 (PMC2474847; doi:10.1186/1742-4690-5-57)

**A**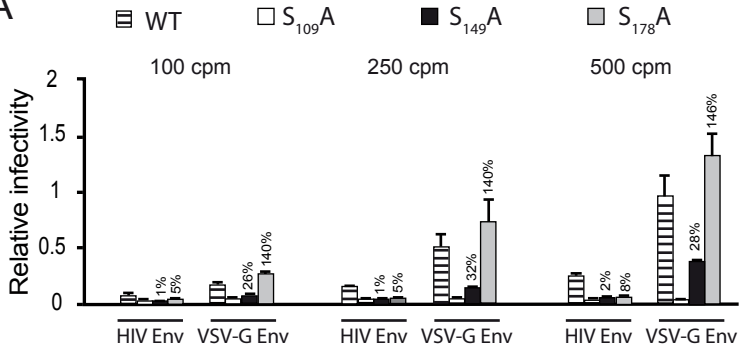**B**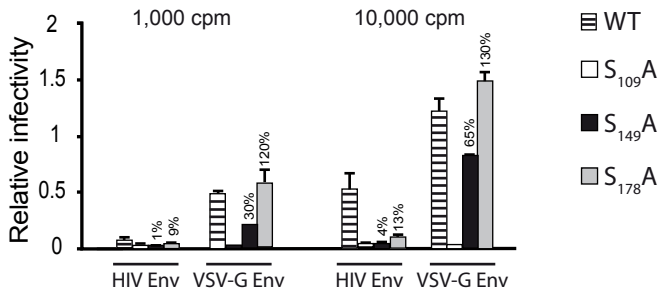

Supplement: Additional file 1 — Infectivity of increasing amounts of WT viruses and CA mutants expressing HIV Env or VSV-G. Viral inputs were normalized according to RTase activity (ranging from 100 to 10,000 cpm) and used to infect MAGIC-5B cells. 48 hours post-infection, relative infectivity was measured by quantification of o-nitrophenyl β-D-galactopyranoside hydrolysis. Time of incubation used for revelation of β-galactosidase assay was adapted to produce non saturating OD at 405 nm (1h45 in panel A and 25 min in panel B). For each dose tested, infectivity of S149A and S178A mutants is indicated as a percentage of β-galactosidase activity generated by an identical amount of WT viruses with the corresponding envelope. [file 1742-4690-5-57-S1.pdf]

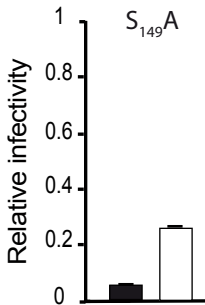

■ HIV Env

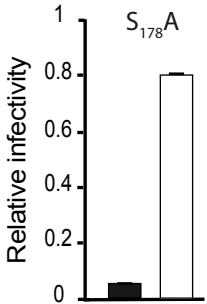

□ VSV-G

Supplement: Additional file 2 — Quantification of β-galactosidase activities generated in MAGIC-5B cells infected with amounts of CA mutants expressing HIV Env or VSV-G that generated comparable strong-stop DNA copy numbers. MAGIC-5B cells were infected with S149A or S178A mutants expressing HIV Env or VSV-G, using infectious doses that generated similar strong-stop DNA copy numbers as measured by qPCR 24 h post-infection. Relative infectivity is expressed as o-nitrophenyl β-D-galactopyranoside hydrolysis measured from total cell extracts by absorbance at 405 nm. Each value represents an average of two experiments performed in duplicate ± standard deviation. [file 1742-4690-5-57-S2.pdf]
